# Medical evacuations by search and rescue helicopters in the Barents Sea

**DOI:** 10.1186/s13049-025-01529-6

**Published:** 2025-12-17

**Authors:** Silje Aune, Torben Wisborg, Hanne Rikstad Iversen, Lasse Raatiniemi

**Affiliations:** 1 Department of Prehospital Services, Finnmark Hospital Trust, Hammerfest, Norway; 2 Department of Anaesthesiology and Intensive Care Medicine, Finnmark Hospital Trust, Hammerfest, Norway; 3 Department of Anaesthesiology and Air Ambulance, University Hospital of North Norway, Tromsø, Norway; 4 Department of Clinical Medicine, UiT, The Artic University of Norway, Tromsø, Norway; 5 Norwegian Centre for Traumatology, Oslo University Hospital, Oslo, Norway

**Keywords:** SAR missions, Medical evacuation, Medevacs, Maritime evacuation, Barents Sea, NACA score

## Abstract

**Background:**

The Barents Sea, characterized by remote locations, vast distances, harsh weather conditions and seasonal polar night, presents significant challenges for search and rescue (SAR) operations. In emergencies involving illness, injury, or other rescue needs, helicopters are often the only feasible response resource. Rescue helicopters are critical for both SAR operations and medical evacuations (medevacs) to meet the region’s urgent demands. The aim of this study was to describe the characteristics of SAR helicopter medevacs in the Barents Sea, with a focus on patient presentations, medical interventions, mission profiles, and operational challenges encountered over a 22-year period.

**Methods:**

A retrospective cohort study reviewed all requests for medevacs involving the SAR helicopter at Banak, operated by the 330 Squadron between 1 January 2000 and 31 December 2022.

**Results:**

A total of 418 requests for medevacs were received, resulting in 283 completed missions. Among the patients 96.5% were male with a median age of 41 years. Most (84%) had a National Advisory Committee for Aeronautics (NACA) score of 3 or 4 on a severity scale from 0 to 7. Wounds and fractures of the upper extremities were the most common injuries, whereas gastrointestinal and cardiovascular conditions were the most frequently reported medical emergencies. Among the trauma cases, 81% were related to the fishing industry. Blunt injuries, including crushing, compression, and pressure-related trauma, were the most common mechanisms. Twenty-five patients (18%) sustained injuries affecting more than one body region.

**Conclusion:**

This study highlights the year-round importance of SAR helicopters in evacuating ill and injured patients from the Barents Sea. Many patients presented with serious but not immediately life-threatening conditions that could deteriorate without timely intervention. These findings reinforce the need for regular and comprehensive training of the entire SAR crew to maintain readiness for patient evacuations in demanding maritime environments.

## Background

The Barents Sea is bordered by mainland Norway to the south, Svalbard to the north, the Norwegian Sea to the west, and Russia to the east. The region is rich in marine life and hosts a significant fishing industry involving multiple nations [1]. In recent years, there has been a marked increase in oil and gas exploration, as well as a rise in cruise tourism, leading to a substantial number of people residing in or travelling through the area at any given time.

In cases of illness, injury, or other emergencies requiring evacuation, helicopters are often the only practical means of response. Rescue operations in this Arctic environment are challenged by remote locations, long distances, extreme weather conditions, and seasonal darkness. Missions are conducted primarily by helicopters stationed at Banak, Tromsø and Longyearbyen.

Limited research has been published on medevacs performed in the Barents Sea. A study using data from ambulance and SAR missions conducted by the 330 Squadron between 1994 and 1999 was published in 2004 and provided an overview of these operations, concluding that they were carried out with high regularity and were in most cases medically justified relative to the operational risk [2]. Given the substantial changes in regional activity and the passage of time, it is essential to reassess mission characteristics and outcomes in a more recent period. Understanding the nature of these missions, including common diagnoses, operational challenges and interventions is crucial for operational planning and resource allocation. Despite a well-established national system for SAR and medevac operations in Norway, little has been published on their medical aspects. Approximately 50 physicians work in these services, providing advanced prehospital care across a wide range of missions. More comprehensive data could support quality improvement and strengthen the future training of medical personnel.

The aim of this study is to investigate medevacs conducted in the Barents Sea from 2000 to 2022. Specifically, we aim to describe the characteristics of the missions, including medical conditions, treatments administered, time intervals, and hoisting methods used.

## Methods

### Study setting

The Royal Norwegian Air Force (RNoAF) 330 Squadron operates six Search and Rescue (SAR) helicopter bases across Norway, with the Banak base, located at 70°N, being the northernmost. In February 2022, the squadron transitioned to AW101 all-weather rescue helicopters, replacing the previous Sea King helicopters. Each SAR helicopter crew consists of six members: two pilots, a hoist operator, a systems operator/navigator, a rescue swimmer (RS), and a consultant physician specialized in anaesthesiology and critical care medicine [3]. Approximately 25–30% of the squadron's missions involve sea rescue operations [4].

The Air Force’s SAR service has search and rescue as its primary mission but also serves as a secondary resource within the national air ambulance service [5].

Emergency medical calls in the Barents Sea region are coordinated by JRCC Northern Norway [6]. Contact may be initiated through the Emergency Medical Communication Centres (EMCC) or directly with the JRCC. Additionally, Radio Medico, operated by the Norwegian Centre for Maritime Medicine and Diving, provides 24/7 medical consultation services to ships and offshore installations [7]. Finally, the physician working in SAR helicopter assesses the need for medevac.

### Clinical governance

The overall medical responsibility for the Banak Search and Rescue (SAR) helicopter service lies with Finnmark Hospital Trust. All clinical procedures adhere to protocols issued by local and regional health authority. Each mission is routinely discussed among the SAR helicopter crew after completion, and the medical lead at the base retrospectively reviews the documentation for all cases. Continuous clinical oversight is further ensured through one to two annual medical review meetings for all physicians at the base. In addition, weekly regional air ambulance meetings are held, where all bases participate to present and discuss recent missions together with representatives from the dispatch centre and the University Hospital of Northern Norway. This governance framework provides both local and regional oversight, ensuring quality, learning, and alignment with national standards.

### Capabilities of the medical crew

The medical crew consists of a physician, who is required to be a specialist in anaesthesiology and intensive care medicine, and a rescue swimmer. In the Norwegian search and rescue system, the rescue swimmer must have a professional background as a registered nurse, paramedic, or ambulance worker, in addition to advanced training in rescue operations [3].

Regular training is mandatory, including recurrent courses in emergency procedures and live tissue training for both professional groups. Physicians are also required to maintain regular on-call duties at the hospital to ensure sufficient exposure to a wide range of procedures and to sustain in-hospital clinical competence. Rescue swimmers are also offered regular hospital placements to maintain clinical skills. This structured framework ensures that all crew members maintain the necessary skills to deliver high-quality prehospital emergency care in a demanding operational environment.

In terms of medical capabilities, the SAR helicopter is equipped to provide advanced critical care in the prehospital setting. The crew has full capacity for anaesthesia, including induction, airway management, and endotracheal intubation. Two ventilators and two patient monitors allow for simultaneous monitoring and treatment of two patients. Analgesia and fluid resuscitation are routinely provided. Red blood cells (RBC) were introduced at the base in autumn 2020, and since 2021 the service has routinely carried two units of whole blood, supplemented with freeze-dried plasma and coagulation factors for haemostatic resuscitation. Broad-spectrum antibiotics and vasopressors are available for the management of sepsis and circulatory shock. The equipment further includes portable ultrasound, a mechanical chest compression device, and the possibility to administer prehospital thrombolysis. Together, these resources enable the crew to deliver advanced life-supporting interventions in challenging maritime and Arctic conditions.

### Management of time-critical conditions in the Barents Sea region

The geographical characteristics of the Barents Sea region involve long transport distances and frequently challenging conditions, which often require treatment and stabilization during transport. The University Hospital of Northern Norway (UNN) in Tromsø is the nearest university hospital and trauma centre, and it serves as the primary referral destination for major trauma, head injuries, acute myocardial infarction as well as endovascular treatment in stroke and severe hypothermia requiring advanced treatments such as extracorporeal membrane oxygenation (ECMO). For patients with ST-elevation myocardial infarction (STEMI), prehospital thrombolysis constitutes the preferred initial reperfusion strategy, as no patient can realistically reach a percutaneous coronary intervention (PCI) center within the recommended timelines.

### Study design

We conducted a retrospective observational study to analyze the characteristics of medevacs performed in the Barents Sea.

### Data source

The study included all requests for medevacs involving the SAR helicopter stationed at Banak from 1 January 2000 to 31 December 2022. Only missions over maritime areas were included.

Search and rescue missions were operationally defined as missions involving hoisting. Only missions in which the primary indication was medevac were included. Initiated missions which were aborted before or during flight were also included in the review.

Data were retrieved from the digital air ambulance record system, LABAS (Labas 7, Norman IT, Norway), which is maintained at the helicopter base. All requests for assistance are recorded systematically in this electronic database by the physician on call.

### Data extraction

The data extracted included patient demographics, diagnosis classified using the International Classification of Diseases (ICD), severity of condition assessed using the National Advisory Committee for Aeronautics (NACA) scale [8], interventions performed and medications administered both on-scene and during transport.

The operational variables included the year and month of the mission, mission irregularities, mission time intervals, pickup positions, rescue techniques, and receiving hospitals.

In cases where pick-up positions were incomplete or missing in the LABAS documentation, coordinates were obtained from the JRCC based on the ship’s reported position at the time of the rescue request.

### Definitions

A rejected mission is defined as one not initiated due to a lack of medical necessity, technical issues before take-off or unfavourable weather conditions. Aborted missions are those cancelled after take-off.

### Data analysis

The data were exported to Microsoft Excel 2019 MSO (version 1808, MS365) for descriptive statistical analysis.

### Ethics and permissions

The Regional Committee for Medical and Health Research Ethics (REK) concluded that the project was not subject to mandatory ethical review (15 June 2023). The study was registered as a research and quality improvement project with the Data Protection Officer at Finnmark Hospital Trust who approved the project and handled the personal data (25 Oct 2023). An exemption from the duty of confidentiality was granted by the Norwegian Directorate of Health permitting access to relevant LABAS records for the specified study period (8 Sept 2023).

## Results

### Mission outcomes

**Fig. 1:**
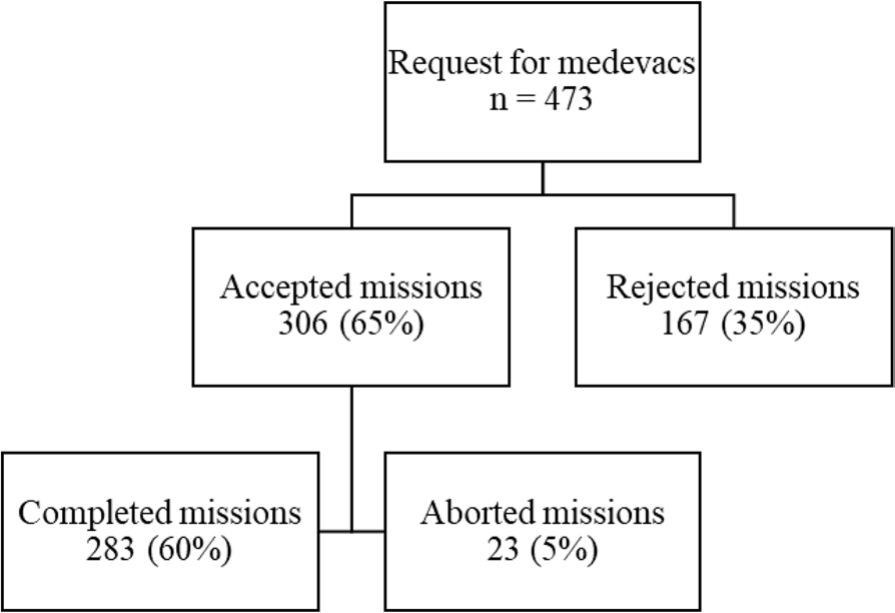
Study diagram showing total requests and completed missions from 2000–2022

During the study period, 473 requests for medevacs by the SAR helicopter stationed at Banak were received for patients located on vessels or offshore installations within the Barents Sea. A total of 283 missions were completed (Fig. 1). The reasons for rejected or aborted missions are presented in Table 1. In some cases, patients had already received advanced medical treatment onboard prior to evacuation, highlighting the capability of certain vessels such as large cruise ships to provide a high level of care

**Table 1:** Reasons for rejected or aborted missions

	**Rejected**	**Aborted**
No need	111	6
Coordination	37	0
Weather	7	5
Deceased	4	3
Technical	2	3
Duty time	2	0
Concurrency conflict	1	3
Other/Not specified	3	3
Total	167	23

Rejected: mission denied before take off

Aborted: mission aborted after take off

No need = the physician assessed that no evacuation was required by SAR helicopter, either prior to deployment or during the mission

Coordination = the mission was resolved by other resources after coordination

Weather = adverse conditions prevented the mission

Deceased = the patient was confirmed dead prior to or during the evacuation attempt

Technical = technical issues with the helicopter prevented completion

Duty time = the mission could not be completed within crew duty regulations

Concurrency conflict = another mission was prioritised and resources were unavailable

Other/Not specified = reason did not fit predefined categories or was not recorded

### Patient characteristics

**Fig. 2:**
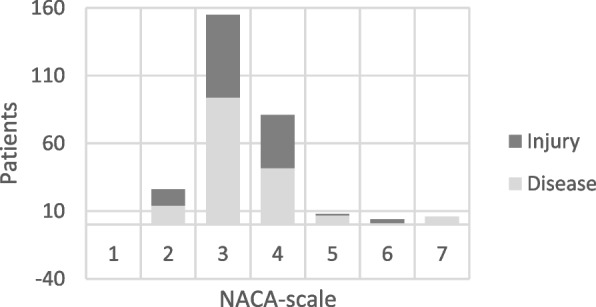
Severity scoring of injury or illness according to the National Advisory Committee for Aeronautics (NACA) for 283 medevacs in the Barents Sea during the period 2000–2022. NACA 0: no injury or disease. NACA 1: injuries/diseases without any need for acute physician care. NACA 2: injuries/diseases requiring examination and therapy by a physician, but hospital admission is not indicated. NACA 3: injuries/diseases without acute threat to life but requiring hospital admission. NACA 4: injuries/diseases that can possibly lead to deterioration of vital signs. NACA 5: injuries/diseases with acute threat to life. NACA 6: injuries/diseases transported after successful resuscitation of vital signs. NACA 7: lethal injuries or diseases (with or without resuscitation attempts)

The majority of patients were male (*n* = 273, 96.5%), while 10 (3.5%) were female. The average age was 41 years, with a range from 17 to 85 years. Medical conditions accounted for 60% (169 cases) of medevac requests, and trauma for the remaining 40% (113 cases). A NACA score of 3 was recorded for 55% of the patients, and 29% were assigned a NACA score of 4 (Fig. 2). Five individuals were declared deceased onboard the vessel prior to evacuation. One patient was hoisted during cardiac arrest while receiving cardiopulmonary resuscitation (CPR). No patients died during helicopter transport. The patients represented 21 different nationalities, with the majority being foreign nationals (53%), of whom the largest group were from Russia, comprising 82 patients (29%). A total of 132 (47%) were Norwegian.

### Mission characteristics

Among trauma-related cases, fishing was the most common activity at the time of injury (*n* = 82, 81%). Crush and compression injuries were the leading mechanisms (Table 2). Upper extremity injuries were most common, typically involving wounds or fractures. For medical emergencies, gastrointestinal and cardiovascular conditions were most frequent. (Table 3).

**Table 2:** Overview of activity at the time of injury, mechanism of injury, affected anatomical regions, and type of injury for 114 traumatic cases

Activity	n	%
Fishing	92	81
Work offshore	11	10
Military service	4	4
Tourism/Leisure	1	1
Not specified	6	5
Mechanism		
Crushed, Compressed, or Pressed	32	30
Blow, Impact, or Collision	22	21
Fall Accidents	17	16
Transport Accident at Sea	12	11
Stabbed, Pinched, Weapons, or Explosion	11	10
Other	13	12
Anatomic region primar diagnosis		
Shoulder/arm/hand	40	35
Lower Extremities	27	24
Head	18	16
Abdomen/back/pelvis	12	11
Eye	7	6
Thorax/Chest	7	6
Other	3	3
Consequence		
Multiple Injuries	25	18
Unspecified	22	16
Wound Injury	19	14
Fracture	17	12
Crush Injury	16	12
Amputation	12	9
Commotio	10	7
Organ Damage	9	7
Other	9	7

Some patients sustained more than one injury or consequence, which explains totals above the number of trauma patients

**Table 3:** Main medical diagnoses among evacuated patients with non-traumatic conditions

Medical diagnosis	n	%
Gastrointestinal	64	38
Cardiovascular	46	27
Neurological	22	13
Diabetes/Embolism/Other	10	6
Urinary tract	9	5
Airways	8	5
Infectious	5	3
Musculoskeletal	5	3

### Medical interventions

The most commonly administered treatments were infusions (*n* = 155), analgesics (*n* = 144), and oxygen therapy (*n* = 116) (Table 4). A total of eight patients underwent endotracheal intubation. In cases of cardiac arrest, endotracheal intubation was performed in four cases. Three of these patients were intubated onboard the boat but were not evacuated due to unsuccessful resuscitation efforts. One patient was hoisted during ongoing CPR and intubated upon arrival in the helicopter. Among the drug-assisted intubations, two was performed on board vessels prior to evacuation, one in-flight and one at the airport before transfer to a fixed-wing aircraft.

**Table 4:** Medical interventions performed during missions

Treatment given to diseased or injured patients	n
Infusion	155
Analgesics	144
Oxygen Therapy	116
Antibiotics	42
Vasoactive Agents	20
Splinting/Traction Devices	14
Endotracheal Intubation	8
Wound Care/Dressings	7
Cardiopulmonary Resuscitation (CPR)	5

One patient could receive more than one intervention, therefore the total number of interventions exceeds the number of patients evacuated

#### Operational conditions

**Fig. 3:**
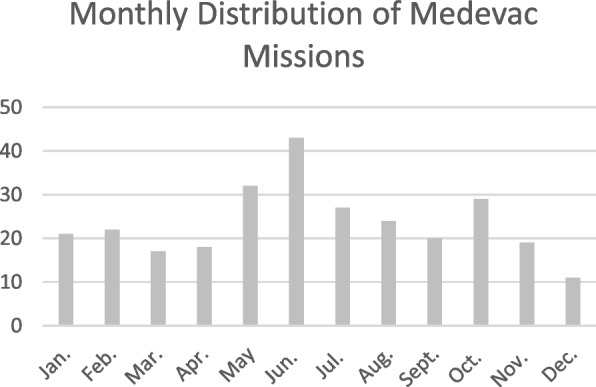
Monthly distribution of missions

**Fig. 4:**
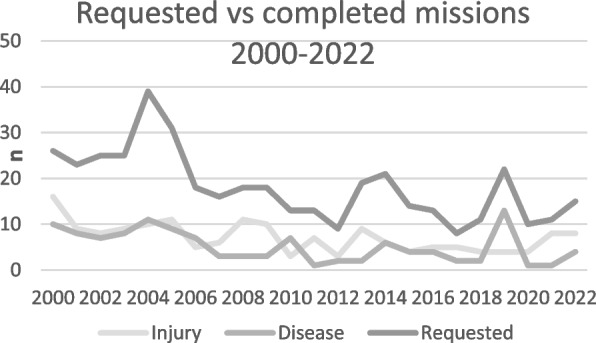
Annual number of requested and completed medevacs in the Barents Sea (2000–2022)

The monthly and annual distributions of missions are presented in Figs. 3 and 4, respectively. A seasonal pattern was observed, with the highest number of missions occurring in June and the lowest number occurring in December. The number of missions decreased over time, from 26 missions in 2000 to 12 missions in 2022.

**Fig. 5:**
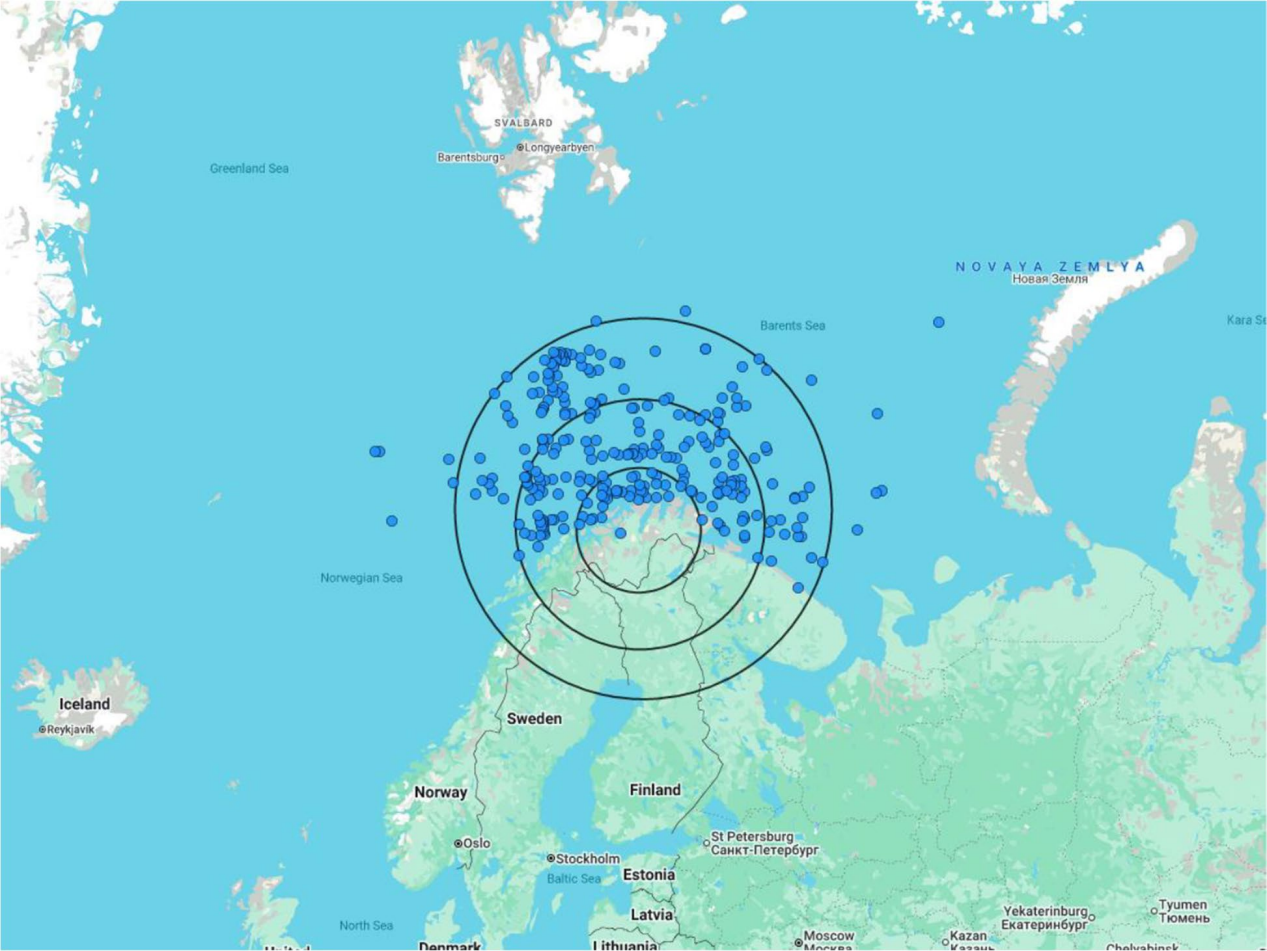
Pick-up locations of medevacs in the Barents Sea. The range rings illustrate distances from the helicopter base at intervals of 100 nautical miles (185 km), 200 nautical miles (370 km), and 300 nautical miles (555 km). Each dot represents the position of å medevac

The pick-up locations are shown in Fig. 5, covering an area from 69.3°N to 74.5°N latitude and from 10.0°E to 42.18°E longitude. The median total mission duration was 332 min (range: 78–1880 min). The total mission time encompasses the entire operation, from scramble to mission completion, including the time required to prepare the helicopter and equipment for subsequent missions. The median response time, defined as the interval from alarm activation to arrival at the patient's location, was 153 min (range: 46–1335 min). The median transport time was 85 min (range: 10–335 min), while the median patient care time was 98 min (range: 10–480 min). Patient care time includes the duration spent attending to the patient, encompassing care at the scene, during transport, and at the destination, until the patient is handed over to the next care provider.

### Rescue techniques

Vertical sling hoisting was the most common method of patient evacuation from vessels (*n* = 139, 49%), followed by horizontal stretcher hoisting (*n* = 102, 36%). In five missions, the helicopter landed directly on the vessel's deck. In five additional missions, a rendezvous was conducted at Bjørnøya. In one mission, the physician was delivered to the vessel and accompanied the boat to land. Five were declared deceased before helicopter transport and were not transported by helicopter.

### Hospital admissions

Most patients (70%) were admitted to local hospitals. 28% was transported directly to the University Hospital of North Norway. Three patients (1%) were transferred to fixed-wing aircraft for onward transport to specialised centres in southern Norway.

## Discussion

Medevacs in the Barents Sea between 2000 and 2022 predominantly involved men of working age. Many patients presented with serious, though not always immediately life-threatening conditions that could have deteriorated without evacuation. Missions often lasted several hours, requiring extended patient care during transport. These findings underline the importance of having trained medical personnel and adequately equipped helicopters capable of managing complex conditions and potential complications en route. Regular training is important to sustain preparedness among medical and flight crews, particularly given the relatively low number of maritime missions per year.

Forty percent of the evacuations involved traumatic injuries, while sixty percent involved medical conditions, which is consistent with previous findings [2, 9]. Traumatic crush injuries to the extremities were the most common type of trauma, with 81% of trauma patients engaged in fishing-related activities. This highlights the key role of SAR services in supporting maritime occupational safety. Given the fishing industry’s importance to Norway, supporting this workforce has both humanitarian and economic implications [10]. Among medevacs, gastrointestinal conditions were most common, followed by cardiovascular events. This contrasts with more accessible areas such as mainland Norway, where cardiovascular conditions typically dominate [1, 11–13]. As reported in Haagensen’s study [2], gastrointestinal conditions were the most frequent cause of evacuation in the Barents Sea. The severe conditions and long distances in the region mean that helicopter transport is often required even when the patient is not critically ill. This underscores the role of SAR operations in supporting emergency care in this remote environment.

Treatment patterns were similar to earlier findings [14, 15], with infusions, analgesics, and oxygen as the most common interventions. Hoisting of intubated patients occurred only twice in 22 years, which aligns with previous studies [14, 16, 17]. Despite a noticeable decline in the total number of missions and a low number of hoist operations per crew member over two decades, the need for regular training remains clear. Thirty-three percent of all patients had a NACA score of 4–6, indicating potentially serious conditions. Even though the annual volume was modest, many required evacuation to prevent deterioration. The fact that all evacuated patients were admitted to hospital suggests that most missions were medically justified. Most evacuations resulted in admission to local hospitals, mainly Hammerfest Hospital, which enhanced logistical efficiency and reduced the burden on tertiary centres.

The distribution of mission outcomes also reflects elements of clinical governance in the service. The large proportion of missions categorised as “no need” indicates that medical assessment, both prior to and during deployment, functions as an important mechanism to avoid unnecessary evacuations. Similarly, cases rejected due to coordination show how structured decision-making with other resources contributes to appropriate use of helicopter capacity. That all evacuated patients were admitted to hospital may indicate consistency in triage and decision-making, in line with regional protocols.

Prehospital emergency care in the Barents Sea region is organized within a joint governance framework, with regular training and close collaboration with hospital specialists. Defined pathways for time-critical conditions (trauma, myocardial infarction, stroke, hypothermia and sepsis) provide a structured basis for treatment, which in the Barents Sea region must be applied in the context of long distances and adverse weather. Measures such as prehospital thrombolysis for STEMI, telemedical consultation for suspected stroke, and the availability of whole blood and antibiotics illustrate how national and regional protocols are implemented under Arctic conditions. Of the total missions, 77 were rejected, primarily due to lack of medical necessity, underlining the potential benefit of early medical consultation for improving resource allocation and coordination of alternatives.

In addition to the clinical and operational implications discussed above, the findings also have relevance for wider emergency preparedness in the region. Beyond individual patient outcomes, they illustrate how the SAR helicopter functions as a crucial resource in northern Norway and as a shared asset for maritime emergencies. This multidisciplinary capability contributes to system resilience in a region with limited redundancy. In this perspective, the SAR helicopter should be regarded as an important component of national total preparedness, complementing its role in patient care.

### Limitations

This study is based on retrospective data manually recorded in the LABAS system by helicopter physicians after the missions, which carries a risk of missing or incomplete entries. An electronic patient record system capable of capturing data automatically during missions could have mitigated this potential bias. Coordinates from JRCC were used to supplement missing pick-up positions; these data often reflect the ship’s location at the time of the distress call, not necessarily the exact evacuation site. We lacked follow-up data after hospital admission, which could have confirmed diagnoses, assessed intervention outcomes, and evaluated the appropriateness of evacuations. The relatively small number of missions per year limits the statistical power of subgroup analyses, and the findings are context-specific for the Barents Sea. They may not be directly generalisable to other regions with different geography, infrastructure, or SAR organisation.

## Conclusion

This study provides a description of the organization and operation of prehospital emergency care in the Barents Sea region over more than two decades. The findings illustrate injury patterns, operational challenges, and medical interventions, and show how clinical governance, crew competence, and structured pathways for time-critical conditions support service delivery in a demanding Arctic context. The results should be interpreted in light of operational limitations, including missions rejected or aborted due to weather, technical factors, or lack of medical need. These constraints underline the challenges of providing advanced emergency care in remote areas and emphasize the importance of robust systems for training, coordination, and preparedness, as well as the potential for future research and operational adjustments to further strengthen safety and efficiency.

## Data Availability

The data used during the current study are available from the corresponding author on reasonable request.

## Abbreviations

CPR: Cardiopulmonary Resuscitation
EMCC: Emergency Medical Communication Centres
ICD: International Classification of Diseases
JRCC: Joint Rescue Coordination Centres
LABAS: Digital Air Ambulance Record System
NACA: National Advisory Committee for Aeronautics
RNoAF: The Royal Norwegian Air Force
SAR: Search And Rescue
